# Supplementary material for: DMRforPairs: identifying Differentially Methylated Regions between unique samples using array based methylation profiles
Source: BMC Bioinformatics. 2014 May 15;15:141. doi: 10.1186/1471-2105-15-141 (PMC4046028; doi:10.1186/1471-2105-15-141)
Supplement: Additional file 2 — DMRforPairs output for the comparison of A431-MCF7 and NA17018-NA17105. Please start from the HTML files in each folder. Available via the BMC Bioinformatics website. [file 1471-2105-15-141-S2.zip › 1394847754114233_MOESM2_ESM/NA17105M_NA17018F/figures/34803.pdf]

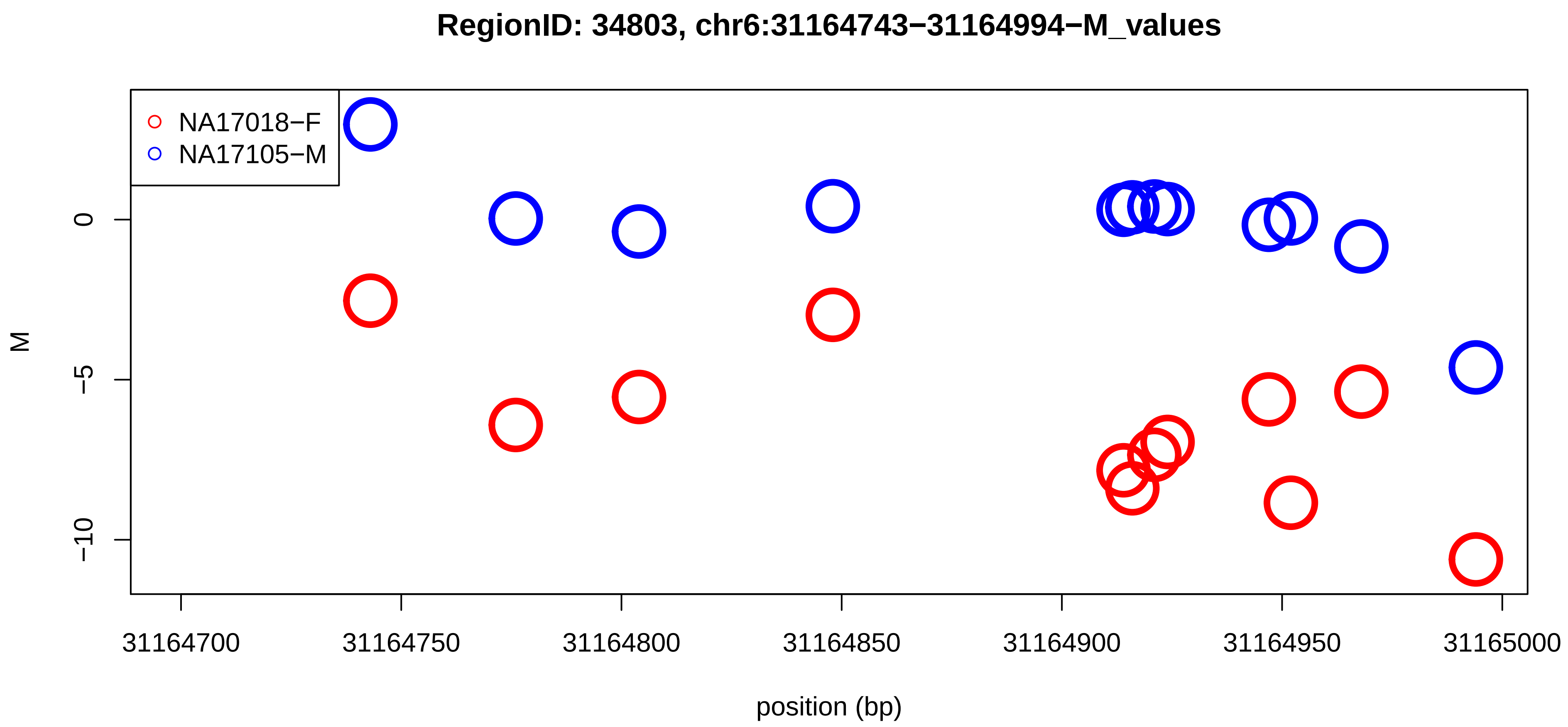

RegionID: 34803, chr6:31164743−31164994−M_values
NA17018−F
NA17105−M
M
0
−5
−10
31164700
31164750
31164800
31164850
31164900
31164950
31165000
position (bp)

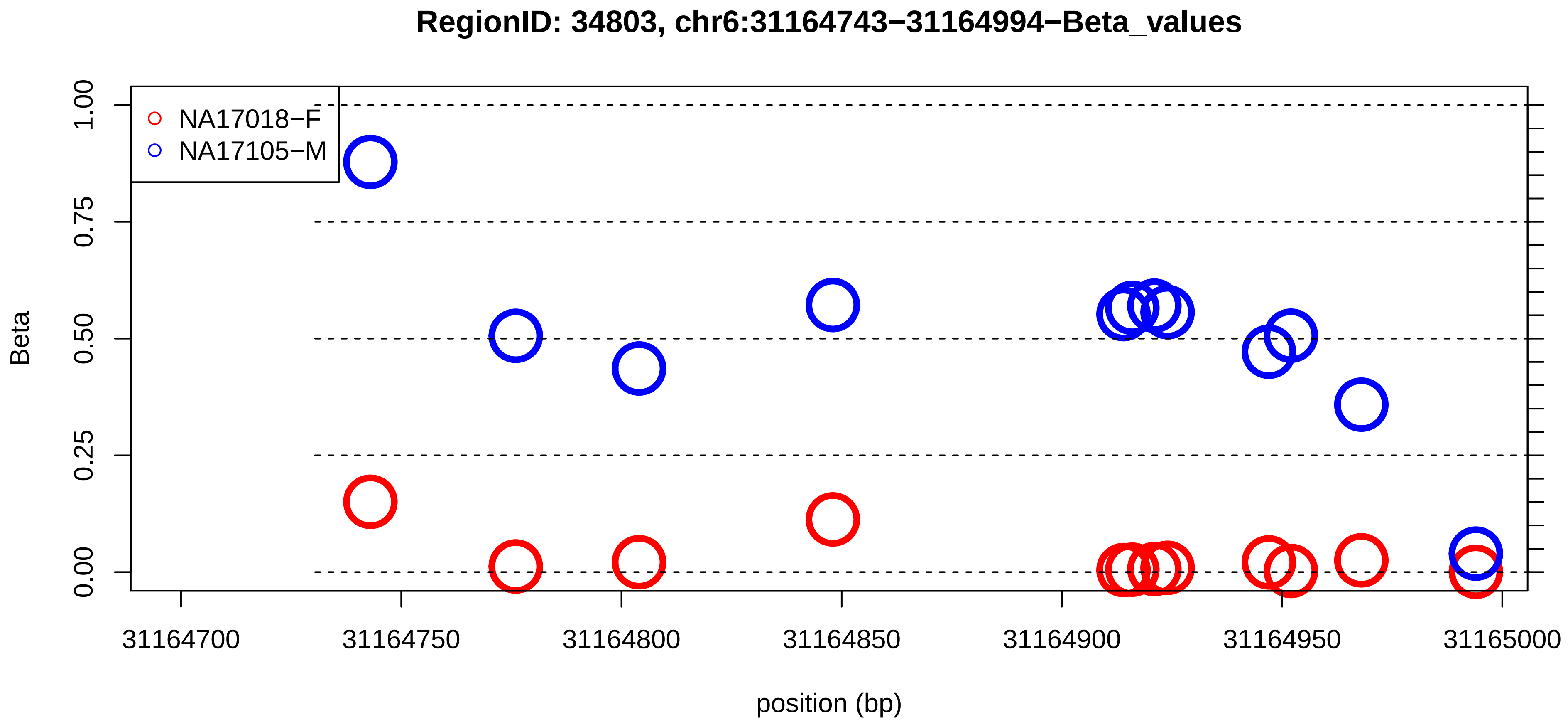

RegionID: 34803, chr6:31164743−31164994−Beta_values
NA17018−F
NA17105−M
Beta
1.00
0.75
0.50
0.25
0.00
31164700
31164750
31164800
31164850
31164900
31164950
31165000
position (bp)

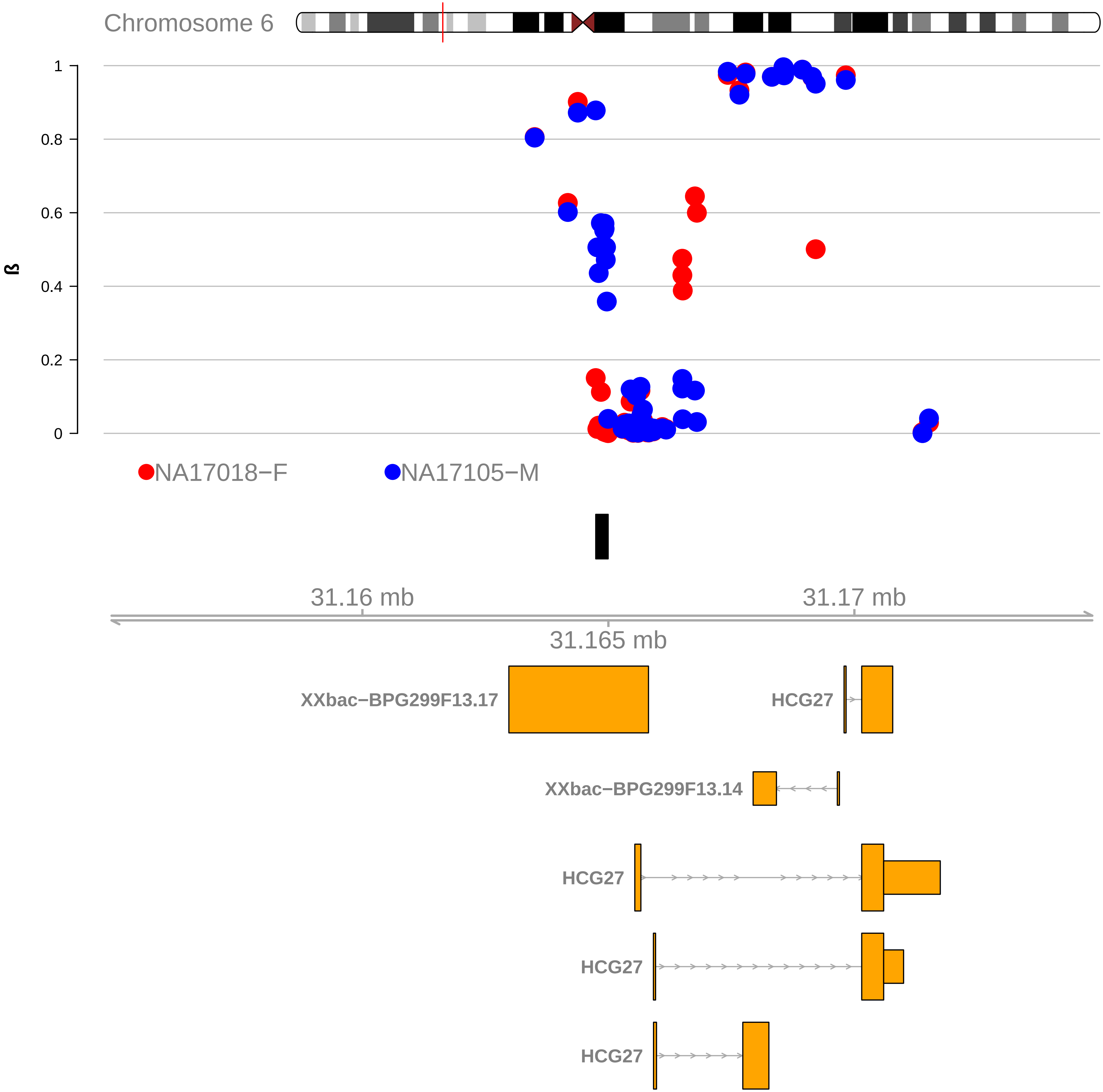
Chromosome 6
ß
1
0.8
0.6
0.4
0.2
0
NA17018−F
NA17105−M
31.16 mb
31.17 mb
31.165 mb
XXbac−BPG299F13.17
HCG27
XXbac−BPG299F13.14
HCG27
HCG27
HCG27
